# The mitochondrial genome of *Hybos serratus* (Diptera: Empididae)

**DOI:** 10.1080/23802359.2018.1501324

**Published:** 2018-10-25

**Authors:** Shang Gao, Junhua Zhang, Ding Yang

**Affiliations:** a College of Plant Protection, China Agricultural University, Beijing, China;; b Institute of Plant Quarantine, Chinese Academy of Inspection and Quarantine, Beijing, China

**Keywords:** Mitochondrial genome, hybotinae, phylogenetics

## Abstract

The dance fly *Hybos serratus* Yang et Yang belongs to the subfamily Hybotinae of Empididae. The mitogenome of *H. serratus* was sequenced, the first representative of the mitogenome of the subfamily. The nearly complete mitogenome is 15,997 bp totally, consisting of 13 protein-coding genes, two rRNAs and 22 transfer RNAs. All genes have the similar locations and strands with that of other published species of Empididae. The nucleotide composition biases towards A and T, which together made up 79% of the entirety. Bayesian inference analysis strongly supported the monophyly of Empidoidea and Dolichopodidae. This result also suggested that Hybotinae is assigned to the sister group to the clade of Dolichopodidae and Empididae.

## Introduction

Empididae is one of the largest families in Diptera with over 5000 described species from the world (Yang et al. 2007). They capture aphids, psyllids and coccids of Hemiptera, but also other true flies such as mosquitos, blackflies and so on. They are widely used as a biological indicator of evaluating the quality of environment and biodiversity (Yang and Yang 2003).

The specimens of *Hybos. serratus* used for this study were collected in Zhouzhi County of Shaanxi Province by Xuankun Li, and identified by Ding Yang. Specimens are deposited in the Entomological Museum of China Agricultural University (CAU). The total genomic DNA was extracted from the whole body (except head) of the specimen using the QIAamp DNA Blood Mini Kit (Qiagen, Germany) and stored at –20 °C until needed. The mitogenome was amplified and sequenced as described in our previous study (Wang et al. 2016a, 2016b, 2016c). The nearly complete mitogenome of *H. serratus* is 15,997bp. It encoded 13 PCGs, 22 tRNA genes, and two rRNA genes and the control region could not be sequenced entirely in this study, and were similar with related reports before (Kang et al. 2016; Li et al. 2016; Wang et al. 2016a, 2016c; Li et al. 2017; Zhou et al. 2017; Gao et al. 2018; Qilemoge et al. 2018). All genes have the similar locations and strands with that of other published Empididae species. The nucleotide composition of the mitogenome was biased towards A and T, with 79% of A + T content (A = 40.3%, T = 38.7%, C = 12.5%, G = 8.5%). The A + T content of PCGs, tRNAs, and rRNAs is 76.8%, 78.8%, and 82.2%, respectively. The total length of all 13 PCGs of *H. serratus* is 11,192 bp. Five PCGs (*ATP8*, *NAD2*, *NAD3, NAD5*, and *NAD6*) initiated with ATT codons, and five PCGs (*COII*, *COIII*, *ATP6*, *NAD4*, and *CYTB*) initiated with ATG codons, *NAD4L* initiated with ATA as a start codon, *CO1* and *NAD1* initiated with TCG and TTG as a start codon, respectively. Eleven PCGs used the typical termination codons TAA, two PCGs (*CO1* and *NAD5*) used T in *H. serratus*.

**Figure 1.**
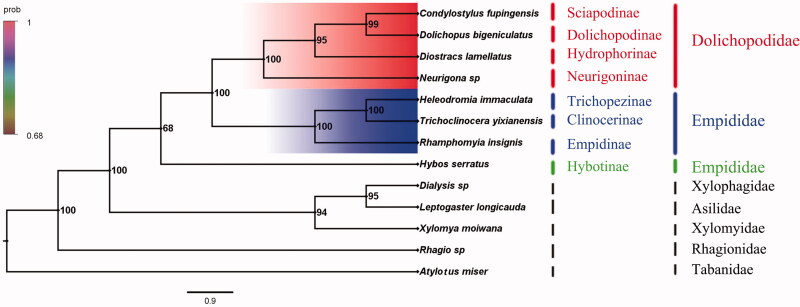
Bayesian phylogenetic tree of 13 Diptera species. The posterior probabilities are labelled at each node.

Phylogenetic analysis was performed based on the nucleotide sequences of 13 PCGs from 13 Diptera species. Bayesian inference (BI) analysis generated the phylogenetic tree topologies based on the PCGs matrices (Figure 1). According to the phylogenetic result, the monophyly of Empidoidea was strongly supported. The monophyletic Dolichopodidae was assigned to the sister to the clade of Empididae that consists of Empidinae, Clinocerinae, and Trichopezinae in this study. For the phylogeny of Empididae, the Empidinae is the sister group to the clade of Clinocerinae + Trichopezinae, but the Hybotinae is the sister group to the clade of Dolichopodidae and Empididae. This result about the classification status of Hybotinae is consistent with the phylogenetic result of previous studies (Wahlberg and Johanson 2018). The mitogenome of *H. serratus* could provide the important information for the further studies of Empidoidea phylogeny.
